# *Burkholderia mallei* expresses a unique lipopolysaccharide mixture that is a potent activator of human Toll-like receptor 4 complexes

**DOI:** 10.1111/j.1365-2958.2006.05519.x

**Published:** 2007-01-01

**Authors:** Paul J Brett, Mary N Burtnick, D Scott Snyder, Jeffrey G Shannon, Parastoo Azadi, Frank C Gherardini

**Affiliations:** 1 Laboratory of Zoonotic Pathogens, Rocky Mountain Laboratories NIAID, NIH, Hamilton, MT 59840, USA.; 2 Complex Carbohydrate Research Center, The University of Georgia Athens, GA 30602, USA.; 3 Laboratory of Intracellular Parasites, Rocky Mountain Laboratories NIAID, NIH, Hamilton, MT 59840, USA.

## Abstract

*Burkholderia mallei*, the aetiologic agent of glanders, causes a variety of illnesses in animals and humans ranging from occult infections to acute fulminating septicaemias. To better understand the role of lipopolysaccharide (LPS) in the pathogenesis of these diseases, studies were initiated to characterize the structural and biological properties of lipid A moieties expressed by this organism. Using a combination of chemical analyses and MALDI-TOF mass spectrometry, *B. mallei* was shown to express a heterogeneous mixture of tetra- and penta-acylated lipid A species that were non-stoichiometrically substituted with 4-amino-4-deoxy-arabinose residues. The major penta-acylated species consisted of bisphosphorylated d-glucosamine disaccharide backbones possessing two amide linked 3-hydroxyhexadecanoic acids, two ester linked 3-hydroxytetradecanoic acids [C14:0(3-OH)] and an acyloxyacyl linked tetradecanoic acid, whereas, the major tetra-acylated species possessed all but the 3′-linked C14:0(3-OH) residues. In addition, although devoid of hexa-acylated species, *B. mallei* LPS was shown to be a potent activator of human Toll-like receptor 4 complexes and stimulated human macrophage-like cells (THP-1 and U-937), monocyte-derived macrophages and dendritic cells to produce high levels of TNF-α, IL-6 and RANTES. Based upon these results, it appears that *B. mallei* LPS is likely to play a significant role in the pathogenesis of human disease.

## Introduction

*Burkholderia mallei*, the aetiologic agent of glanders, is a non-motile, aerobic, facultative-intracellular Gram-negative bacillus that is primarily responsible for disease in horses, mules, donkeys and occasionally humans (Howe and Miller, 1947; Redfearn *et al*., 1966; Yabuuchi *et al*., 1992; Srinivasan *et al*., 2001). In horses, glanders presents as chronic or acute illnesses characterized primarily by lung involvement, ulcerative nasal and tracheal lesions and visceral abscess formation. In contrast, farcy, the cutaneous form of the disease, is characterized by the presence of nodules and ulcerative skin lesions on the extremities of the animals (Redfearn *et al*., 1966; Howe *et al*., 1971; Bartlett, 2005). The clinical progression of human glanders is similar to that observed in solipeds and may manifest as chronic or acute localized infections, acute pulmonary infections or fulminating septicaemias. Although rare, human infections are thought to be acquired via the inoculation of mucosal or cutaneous tissues with aerosols or secretions from diseased animals. In the absence of chemotherapeutic intervention human glanders is invariably fatal (Dance, 1996; Bartlett, 2005). Due to this, the Centers for Disease Control and Prevention have listed *B. mallei* as a category B biological threat agent (Rotz *et al*., 2002). Currently, there are no human or veterinary vaccines commercially available for immunization against these diseases.

Although glanders is one of the oldest of known infectious diseases, the molecular mechanisms by which *B. mallei* causes disease remain poorly defined (Wilkinson, 1981). Recently, however, a number of groups have demonstrated that quorum sensing, type three secretion system and capsular polysaccharide (CPS) mutants are attenuated in their ability to cause disease in mice, golden hamsters and/or miniature horses (DeShazer *et al*., 2001; Lopez *et al*., 2003; Ulrich and DeShazer, 2004; Ulrich *et al*., 2004). In addition, we have previously shown that *B. mallei* strains expressing rough lipopolysaccharide (LPS) phenotypes are far more sensitive to the bactericidal effects of normal human serum than are those expressing a smooth phenotype also implicating LPS as potential virulence determinant (Burtnick *et al*., 2002).

Lipopolysaccharides, commonly referred to as endotoxins, are a major component of Gram-negative cell envelopes (Burns *et al*., 2006). The ‘barrier function’ provided by bacterial outer membranes is largely due to the presence of these molecules (Nikaido, 2003). Bacterial strains expressing a ‘smooth’ phenotype synthesize LPS antigens that are composed of three covalently linked domains: an O-antigen, a core region and a lipid A moiety (Raetz and Whitfield, 2002). Lipid A, which normally functions as the hydrophobic membrane-anchor component of LPS, is also the domain responsible for stimulating a variety of pathophysiological responses in mammals (Alexander and Rietschel, 2001; Trent, 2004). In general, bisphosphorylated hexa-acylated lipid A species possessing C12 and C14 fatty acids represent the most biologically active forms of the molecule (Erridge *et al*., 2002; Raetz and Whitfield, 2002; Caroff and Karibian, 2003). Lipid A moieties that deviate from this pattern often demonstrate a significant decrease in endotoxic activity (Alexander and Rietschel, 2001; Backhed *et al*., 2001; 2003).

Host recognition of LPS antigens by mammalian cells is mediated through interactions with Toll-like receptor 4 (TLR4) complexes. These complexes, consisting of TLR4 receptors and the MD2 and CD14 coreceptors enable a variety of cell types to rapidly detect the presence of Gram-negative bacteria in host tissues (Poltorak *et al*., 1998; da Silva Correia *et al*., 2001; Miller *et al*., 2005). Monocytes and macrophages, for instance, when activated by LPS through TLR4 complexes secrete pro-inflammatory cytokines such as TNF-α, IL-1β and IL-6 which augment innate immune responses against invading microbes. In excess, however, these cytokines can cause multiple organ failure, shock and death (Zanotti *et al*., 2002). Although studies have examined some structural aspects of *B. mallei* LPS, none have actually reported on a potential immunopathological role for this molecule (Pitt *et al*., 1992; Anuntagool and Sirisinha, 2002; Woods *et al*., 2002). In order to address this issue, we have utilized a combination of genetic, chemical, physical and immunological approaches to further characterize the LPS expressed by this bacterial pathogen. The main objective of this study was to develop a better understanding of the potential role of endotoxin in the pathogenesis of disease caused by this organism.

## Results

### Characterization of purified LPS antigens

**Fig. 1:**
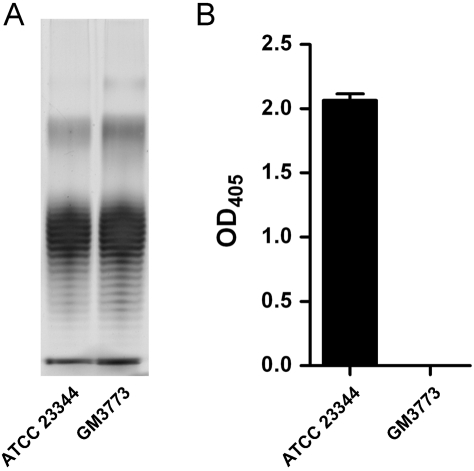
Analysis of purified LPS preparations A. SDS-PAGE. The *B. mallei* ATCC 23344 and GM3773 LPS preparations (1 μg lane^−1^) were electrophoresed on 12% Express Gels and visualized by silver staining. B. CPS-specific ELISA. Microtiter plate wells were coated with the ATCC 23344 or GM3773 LPS preparations (500 ng well^−1^) and then incubated with the MCA147 mAb to assay for the presence of CPS. Error bars represent the standard deviation of samples assayed in quadruplicate. The figure is representative of two independent experiments.

In order to obtain highly purified LPS for use in this study, endotoxin was isolated from *B. mallei* GM3773 (CPS mutant) as well as ATCC 23344 (wild-type strain) using a modified enzyme hot aqueous-phenol procedure. SDS-PAGE and silver stain analysis of the LPS isolated from the CPS mutant demonstrated that the preparation consisted of a mixture of both rough and smooth species. Further examination of the gel revealed no apparent structural differences between the LPS species isolated from the CPS mutant and wild-type strain (Fig. 1A). Similar results were also observed by immunoblot analysis (data not shown). Additional analysis of the samples by enzyme-linked immunosorbent assays (ELISA), however, demonstrated that while the GM3773 preparation contained no detectable levels of the CPS, the LPS isolated from ATCC 23344 was contaminated with this antigen (Fig. 1B). Based upon these findings, as well as those from the chemical and MALDI-TOF-MS analyses (see below), we opted to utilize the LPS isolated from GM3773 to determine the biological activities associated with *B. mallei* endotoxin.

### Composition and chemical analyses of lipid A

Lipid A samples were isolated from the purified LPS preparations via mild acid hydrolysis. Glycosyl composition analysis of lipid A obtained from *B. mallei* GM3773 LPS confirmed the presence of d-glucosamine (GlcN) in the sample. Fatty acid composition analysis of the lipid A sample also revealed the presence of tetradecanoic (C14:0), 3-hydroxytetradecanoic [C14:0(3-OH)], hexadecanoic (C16:0) and 3-hydroxyhexadecanoic acids [C16:0(3-OH)] in the molar ratios of ∼1.0:1.6:0.2:1.9 respectively. In addition, *O*-linked fatty acid analyses demonstrated that the C14:0, C14:0(3-OH) and C16:0 residues were ester linked indicating that the C16:0(3-OH) residues were amide linked. Similar results were also obtained for the lipid A antigens isolated from the *B. mallei* ATCC 23344 LPS.

### MALDI-TOF-MS analyses of lipid A

**Fig. 2:**
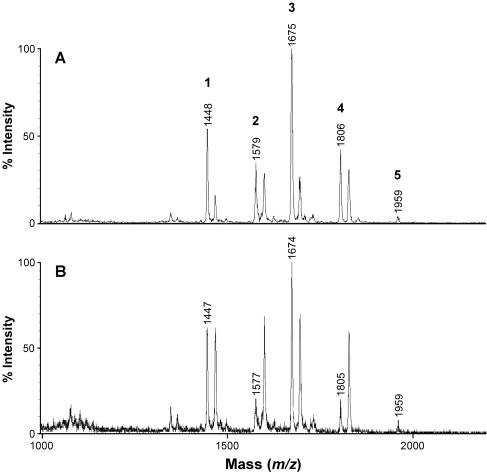
Negative-ion MALDI-TOF mass spectra of lipid A species isolated from purified LPS antigens. (A) *B. mallei* GM3773 and (B) ATCC 23344 mass spectra. The molecular ion peaks (1–5) are described in Table 1 or in the text. Sodium adducts (Δ*m/z* + 22) are not labelled.

The negative-ion MALDI-TOF mass spectrum of the purified *B. mallei* GM3773 lipid A preparation revealed a complex pattern of molecular ion peaks indicative of a heterogeneous mixture of species (Fig. 2A). Utilizing the information obtained from chemical and composition analyses in combination with the MS data, the molecular ion peaks were assigned as follows. The four main series of ion peaks identified were representative of a combination of tetra-acylated (*m/z* 1448 and 1579) and penta-acylated (*m/z* 1675 and 1806) species (Table 1). These species were also present in significant quantities as sodium adducts (Δ*m/z* + 22). The ion at *m/z* 1448 (species 1) was consistent with a tetra-acylated bisphosphorylated GlcN disaccharide backbone possessing C14:0 and C14:0(3-OH) residues in ester linkage and two C16:0(3-OH) residues in amide linkage. The ion at *m/z* 1579 (species 2) was representative of species 1 modified with a 4-amino-4-deoxy-arabinose (Ara4N) residue (Δ*m/z* + 131). The ion at *m/z* 1675 (species 3) was consistent with a penta-acylated bisphosphorylated GlcN disaccharide backbone possessing one C14:0 residue and two C14:0(3-OH) residues in ester linkage and two C16:0(3-OH) residues in amide linkage. The ion at *m/z* 1806 (species 4) was representative of species 3 modified with an Ara4N residue. The minor ion peak at *m/z* 1959 (species 5) was representative of a species 3 sodium adduct modified with two Ara4N residues. Taking into account the results of the fatty acid composition analyses, the GM3773 preparation appeared to contain similar ratios of the tetra- and penta-acylated species. Similar results were obtained for LPS isolated from *B. mallei* ATCC 23344 confirming that lipid A species expressed by the wild type and CPS mutant strains were identical to one another (Fig. 2B).

**Table 1:** Main MALDI-TOF-MS negative-ion peaks and the proposed interpretation of the fatty acid, phosphate and carbohydrate substituents on the *B. mallei* GM3773 lipid A backbone.

Lipid A species^a^	Observed ion (m/z)	Calculated ion (Da)	Acyl substitution	Predicted fatty acid, phosphate and Ara4N composition
1	1448	1445.4	Tetra	C14:0(3-OH), 2 × C16:0(3-OH), C14:0, 2 × P
2	1579	1576.5	Tetra	C14:0(3-OH), 2 × C16:0(3-OH), C14:0, 2 × P, Ara4N
3	1675	1671.3	Penta	2 × C14:0(3-OH), 2 × C16:0(3-OH), C14:0, 2 × P
4	1806	1802.3	Penta	2 × C14:0(3-OH), 2 × C16:0(3-OH), C14:0, 2 × P, Ara4N

a Sodium adducts (Δm/z + 22) are not listed.

**Fig. 3:**
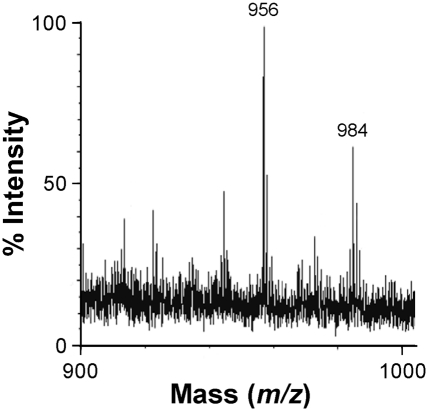
Positive-ion MALDI-TOF mass spectra of lipid A isolated from *B. mallei* GM3773. The significance of the major (*m/z* 956) and minor (*m/z* 984) oxonium ion peaks are described in the text.

**Fig. 4:**
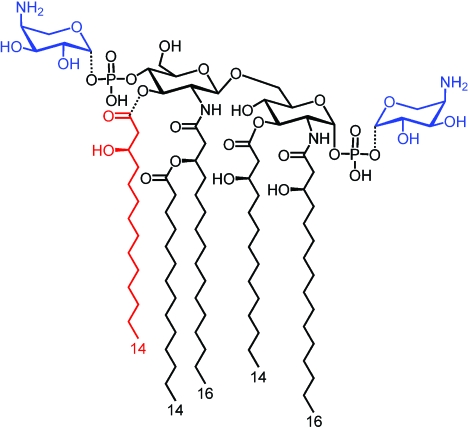
Predicted structures of the major lipid A species identified in this study. Non-stoichiometric substitutions are depicted in blue (Ara4N) and red [C14:0(3-OH)].

The positive-ion MALDI-TOF mass spectrum of the purified *B. mallei* GM3773 lipid A preparation demonstrated the presence of a number of diagnostic oxonium ions (m/z 984, 956, 736 and 730) arising from the cleavage of the glycosidic linkage between the reducing and non-reducing GlcN residues of the lipid A backbone. The major oxonium ion observed at *m/z* 956 was consistent with the sodium adduct of a non-reducing GlcN possessing one ester linked C14:0 residue, one ester linked C14:0(3-OH) residue and one amide linked C16:0(3-OH) residue. In contrast, the minor ion observed at *m/z* 984 was consistent with a non-reducing GlcN possessing one ester linked C16:0 residue, one ester linked C14:0(3-OH) residue and one amide linked C16:0(3-OH) residue (Fig. 3). In addition, the major oxonium ion observed at *m/z* 730 was consistent with the sodium adduct of a non-reducing GlcN possessing an amide linked C16:0(3-OH) residue esterified by a C14:0 residue while the minor ion observed at *m/z* 736 was consistent with a non-reducing GlcN possessing an amide linked C16:0(3-OH) residue esterified by a C16:0 residue (data not shown). Based upon the results of these analyses the proposed structures of the major lipid A species identified in this study are depicted in Fig. 4.

### *Burkholderia mallei* LPS is a potent activator of hTLR4 complexes

**Fig. 5:**
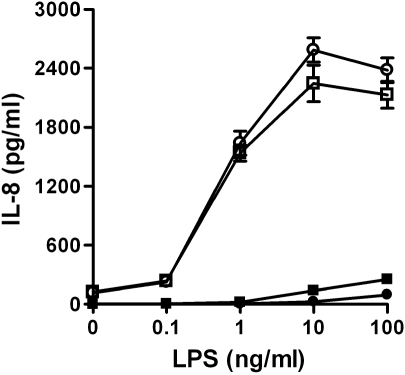
*B. mallei* LPS is a potent hTLR4 agonist. HEK 293-hTLR4/MD2-CD14 cells were stimulated with varying concentrations of *B. mallei* GM3773 (open squares) or *E. coli* O55:B5 (open circles) LPS following which the culture supernatants were assayed for IL-8 production. HEK 293-hTLR2/CD14 were similarly stimulated with *B. mallei* (filled squares) or *E. coli* (filled circles) LPS and the culture supernatants assayed for IL-8 production. Values represent the means ± SD of one experiment assayed in triplicate. The figure is representative of three independent experiments.

Lipopolysaccharide can activate a variety of cell types through interactions with TLR4 complexes (Fujihara *et al*., 2003). The magnitude of cell activation, however, is greatly influenced by endotoxin structure (Backhed *et al*., 2003). In order to characterize the ability of *B. mallei* LPS to function as a hTLR4 agonist, HEK 293 cells expressing hMD2-CD14, hTLR2-CD14 or hTLR4-MD2-CD14 were stimulated with varying concentrations of endotoxin and the culture supernatants were assayed for IL-8. The results of these studies demonstrated that when stimulated with the *B. mallei* LPS, the HEK 293-hTLR4/MD2-CD14 cells produced dose-dependent IL-8 responses that were similar to those observed when stimulated with the *Escherichia coli* O55:B5 LPS control. In contrast, the HEK 293-hTLR2/CD14 cells secreted significantly lower levels of IL-8 when stimulated with identical concentrations of the LPS samples (Fig. 5). In addition, neither of the *E. coli* or *B. mallei* LPS preparations stimulated responses from the HEK 293-hMD2-CD14 cells at any of the concentrations tested (0.1–100 ng ml^−1^; data not shown). Based upon the results of these assays, *B. mallei* LPS was shown to be a potent activator of hTLR4-complexes.

### *Burkholderia mallei* endotoxin activates human macrophages and dendritic cells

**Fig. 6:**
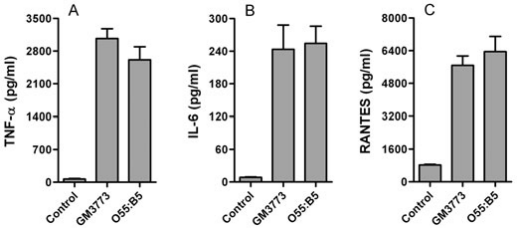
Production of TNF-α, IL-6 and RANTES by LPS stimulated THP-1 cells. THP-1 cells were stimulated for 24 h with 10 ng ml^−1^ of *B. mallei* GM3773 or *E. coli* O55:B5 LPS following which the culture supernatants were assayed for the production of (A) TNF-α (B) IL-6 or (C) RANTES. Unstimulated cells were included as a control. Values represent the means ± SD of four independent experiments assayed in duplicate.

**Fig. 7:**
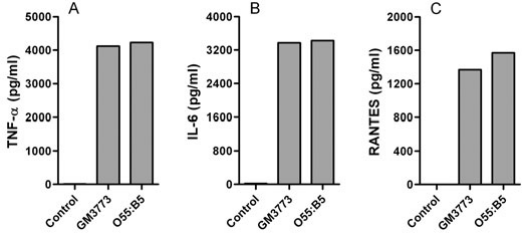
Production of TNF-α, IL-6 and RANTES by LPS stimulated MDM. MDM cells were stimulated for 24 h with 10 ng ml^−1^ of *B. mallei* GM3773 or *E. coli* O55:B5 LPS following which the culture supernatants were assayed for the production of (A) TNF-α (B) IL-6 or (C) RANTES. Unstimulated cells were included as a control. Values represent the means of two readings. The figures are representative of three independent experiments.

**Fig. 8:**
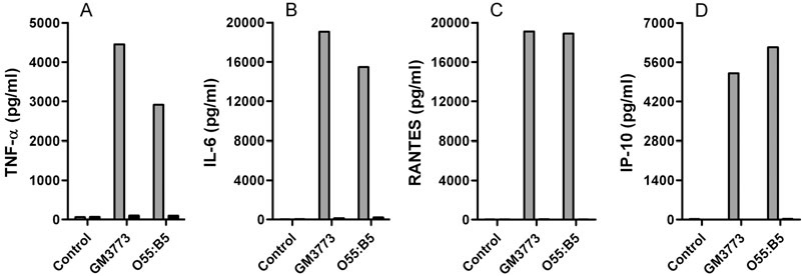
Production of TNF-α, IL-6, RANTES and IP-10 by LPS stimulated MDDC is CD14-Dependent. Prior to stimulation, the MDDC were incubated for 30 min with an anti-CD14 antibody (black bars) or a non-specific isotype control (grey bars). The cells were then stimulated for 24 h with 10 ng ml^−1^ of *B. mallei* GM3773 or *E. coli* O55:B5 LPS following which the culture supernatants were assayed for the production of (A) TNF-α, (B) IL-6, (C) RANTES or (D) IP-10. Unstimulated cells were included as a control. Values represent the means of two readings. The figures are representative of three independent experiments.

Antigen presenting cells (APCs) such as macrophages and dendritic cells recognize bacteria through TLRs and other pattern recognition receptors. Stimulation of APCs through these receptors activates signal transduction cascades that culminate in the production of cytokines, chemokines and other costimulatory molecules. To test the ability of *B. mallei* LPS to activate human APCs, macrophage-like THP-1 cells were stimulated with 10 ng ml^−1^ of LPS isolated from GM3773 and the culture supernatants were assayed for the production of specific cytokines and chemokines. When stimulated with GM3773 LPS, THP-1 cells produced TNF-α, IL-6 and RANTES responses that were similar to those detected using *E. coli* O55:B5 LPS as a control (Fig. 6). Similar trends were also observed when the macrophage-like U-937 cells were stimulated with *B. mallei* LPS (data not shown). In addition, studies demonstrated that when MDM were stimulated with *B. mallei* LPS, they produced high levels of TNF-α, IL-6 and RANTES (Fig. 7). Furthermore, when stimulated with *B. mallei* LPS, MDDC secreted high levels of TNF-α, IL-6, RANTES and IP-10 in a CD14-dependent manner (Fig. 8). Taken together, these results indicated that *B. mallei* LPS was a potent activator of human APCs and was capable of stimulating cytokine and chemokine responses that were consistent with the activation of both hTLR4-MyD88-dependent (TNF-α and IL-6) and -independent (RANTES and IP-10) signalling pathways (Kawai *et al*., 2001; Re and Strominger, 2001; Fitzgerald *et al*., 2003; Zughaier *et al*., 2005).

## Discussion

Lipopolysaccharide antigens expressed by *Burkholderia pseudomallei* and *B. mallei* are typically isolated from whole cell pellets using a modified hot aqueous-phenol extraction procedure (Brett and Woods, 1996; Burtnick *et al*., 2002; Brett *et al*., 2003; Perry *et al*., 1995). Unfortunately, the smooth LPS species expressed by these organisms partition into the phenol phase rather than the aqueous phase following extraction so that isolating homogeneous preparations of these molecules can be a challenge (Perry *et al*., 1995). For example, the high molecular weight CPS expressed by virulent isolates of *B. mallei* and *B. pseudomallei* partitions with the LPS into the phenol phase so that size exclusion chromatography is required to separate these carbohydrate antigens. In order to simplify the task of obtaining highly purified preparations of LPS for use in this study, we constructed *B. mallei* GM3773. By using this CPS mutant, we were able to isolate LPS antigens that were structurally identical to those expressed by the wild-type strain yet were free from any contaminating CPS. In addition, because *B. mallei* CPS mutants are attenuated for virulence (DeShazer *et al*., 2001), working with strain GM3773 minimized exposure to the virulent type strain.

Recent studies by Silipo *et al*. have demonstrated that the human pathogen, *Burkholderia cepacia* BTS7, expresses a complex mixture of tetra- and penta-acylated lipid A species that are non-stoichiometrically substituted with Ara4N residues (Silipo *et al*., 2005). Structural analysis of the lipid A moieties isolated from this organism indicated that the penta-acylated species with the highest mass consisted of disaccharide backbones that were substituted with two phosphoryl-arabinosamine residues [Ara*p*4N-l-β-1-P-4-β-d-Glc*p*N-(1′-6)-α-d-Glc*p*N-1-P-1-β-l-Ara*p*4N] and possessed two ester linked C14:0(3-OH) chains, two amide linked C16:0(3-OH) chains and an acyloxyacyl linked C14:0 residue at the C-3 position of the 2′-C16:0(3-OH) chain. In addition, studies confirmed that the tetra-acylated species with the highest mass were identical to the penta-acylated species except for the lack of the 3′-ester linked C14:0(3-OH) chain. By utilizing a combination of chemical analyses and MALDI-TOF-MS in the present study, we have been able to demonstrate that *B. mallei* also expresses a heterogeneous mixture of tetra- and penta-acylated lipid A species that appear to be non-stoichiometrically substituted with Ara4N residues. In addition, all of the major molecular ion peaks identified in this study were also observed during the analysis of the *B. cepacia* BTS7 lipid A moieties. Interestingly, however, none of the minor *B. mallei* lipid A species predicted to bear acyloxyacyl linked C16:0 residues at the 2′-position that were identified during our studies were shown to be expressed by *B. cepacia* BTS7. At present, the significance of this phenomenon remains unknown.

Previous studies indicate that lipid A antigens consisting of 1,4′-bisphosphorylated β(1′-6)-linked d-glucosamine backbones substituted with two (R)-3-acyloxyacyl and two (R)-3-hydroxyacyl residues in either the 4 + 2 (*E. coli*-like) or 3 + 3 (*Neisseria meningitidis*-like) configuration represent the optimal structures for activating human and mammalian TLR4-complexes (Alexander and Rietschel, 2001; Erridge *et al*., 2002; Dixon and Darveau, 2005; Zughaier *et al*., 2005; 2006). In addition, it has been shown that lipid A antigens deviating from these patterns tend to exhibit reduced biological activities in comparison to these molecules (Kumazawa *et al*., 1988; Nakatsuka *et al*., 1989; Takada and Kotani, 1989; Backhed *et al*., 2003). Consistent with these findings, Bäckhed *et al*. have demonstrated that LPS antigens isolated from wild-type strains of *E. coli* stimulate significantly higher levels of IL-8 from human bladder carcinoma cells (T24 cells) than penta-acylated LPS antigens isolated from *waaN* (*lpxM*) mutants (Backhed *et al*., 2001). Interestingly, Ernst *et al*. have also shown that LPS preparations obtained from *Pseudomonas aeruginosa* clinical isolates expressing both penta- and hexa-acylated species stimulate more robust pro-inflammatory responses from human macrophages and endothelial cells than LPS preparations purified from clinical isolates expressing only penta-acylated species (Ernst *et al*., 1999; 2003; Hajjar *et al*., 2002). In contrast to established structure/function paradigms, however, our studies indicate that although devoid of hexa-acylated species, *B. mallei* LPS is a potent hTLR4 agonist and is capable of stimulating human APCs in a manner similar to *E. coli* LPS. Based upon these results, it appears that by optimizing the length, number and order of their lipid A fatty acids, organisms such as *B. mallei* and *B. cepacia* are able to synthesize a mixture of tetra and penta-acylated LPS antigens whose individual or combined biological properties mimic those typically associated with hexa-acylated LPS preparations (Zughaier *et al*., 1999; De Soyza *et al*., 2004; Bamford *et al*., 2006).

Capping of lipid A phosphates represents an important molecular mechanism utilized by Gram-negative pathogens to resist the bactericidal effects of cationic antimicrobial peptides (CAPs) produced by host innate immune responses (Vaara, 1992; Gunn *et al*., 1998; 2000; Gunn, 2001; Trent, 2004). By modifying lipid A phosphates with amine-containing substituents like Ara4N and phosphoethanolamine, pathogens such as *Salmonella typhimurium* and *Yersinia pestis* can lower their affinity for CAPs and cationic antibiotics by decreasing the overall negative charge of their cell surfaces (Zhou *et al*., 2001; Kawahara *et al*., 2002; Trent, 2004). In *S. typhimurium*, modification of lipid A moieties with Ara4N residues is governed by the PmrA/PmrB two-component system which regulates the expression of genetic loci necessary for the biosynthesis of Ara4N (Soncini and Groisman, 1996). Transcription of PmrA-regulated loci is induced by ferric iron (Fe^+3^), which is sensed by the cognate sensor-PmrB, and by low concentrations of magnesium (Mg^+2^), which requires not only PmrA and PmrB but also PhoP, PhoQ and PmrD (Groisman, 2001; Winfield *et al*., 2005). Consistent with current observations that *B. mallei* lipid A moieties are modified with Ara4N residues, bioinformatics analyses have revealed that a number of genetic loci (*ugd*, *arnA*, *arnB*, *arnC* and *arnT*) known to be involved in the biosynthesis of Ara4N and its transfer to *S. typhimurium* and *E. coli* lipid A phosphates are also present in the genome of *B. mallei* ATCC 23344 (Trent *et al*., 2001; Nierman *et al*., 2004). Curiously, however, although a putative *pmrA* homologue was identified during these analyses, no obvious *pmrB*, *pmrD*, *phoP* or *phoQ* homologues were found. Studies are ongoing in order to characterize the mechanism(s) by which *B. mallei* modifies lipid A moieties with Ara4N residues and the potential significance of these modifications with regards to CAP resistance.

In addition to remodelling the charge characteristics of their LPS antigens, *S. typhimurium*, *P. aeruginosa* and *Y. pestis* are also capable of modifying the acylation patterns of their lipid A moieties in response to environmental changes such as temperature, pH and cation concentrations (Ernst *et al*., 1999; 2006; Zhou *et al*., 2001; Kawahara *et al*., 2002; Rebeil *et al*., 2004; 2006; Tran *et al*., 2005). In particular, modifications resulting in the synthesis of tetra-acylated species have been shown to significantly affect virulence phenotypes as well as play an important role in modulating mammalian TLR4-dependent immune responses (Ernst *et al*., 1999; Hajjar *et al*., 2002; Backhed *et al*., 2003; Rebeil *et al*., 2004). Recently, Rebeil *et al*. demonstrated that when grown at 21°C, *Y. pestis* expressed a heterogeneous mixture of LPS antigens in which hexa-acylated species predominated, while at 37°C, the organism expressed a mixture of LPS species in which tetra-acylated forms of the antigen predominated (Rebeil *et al*., 2004). During the study, it was also shown that LPS preparations isolated from *Y. pestis* grown at 37°C stimulated significantly less TNF-α production from human peripheral blood monocytes than did LPS preparations purified from the organism when grown at 21°C. Based upon these findings it was suggested that the ability of *Y. pestis* to modify LPS acylation patterns in response to changing temperature may enhance its ability to survive during transitions from its flea vector to mammalian hosts. More recently, Reife *et al*. demonstrated that tetra-acylated LPS species expressed by *Porphyromonas gingivalis* are potent TLR4 antagonists and that by altering the relative amounts these antigens in its outer membrane the organism may be capable of modulating host immune responses (Reife *et al*., 2006). Interestingly, studies have shown that LPS preparations from a clinical isolate of *Burkholderia cenocepacia* expressing only penta-acylated species stimulated stronger TNF-α and IL-6 responses from U-937 cells than from LPS preparations purified from a clinical isolate of *Burkholderia multivorans* expressing both tetra- and penta-acylated species (De Soyza *et al*., 2004).

In the present study we have demonstrated that *B. mallei* expresses a mixture of tetra- and penta-acylated LPS species that in combination exhibit some unique biological properties. In order to fully characterize the significance of this heterogeneity, however, further studies will be required to elucidate the specific activities of these individual LPS species. Whether or not changes in environmental conditions/stimuli modulate LPS acylation patterns and biological activity also remains to be experimentally defined. Studies are ongoing to address these important issues.

## Experimental procedures

### Bacterial strains and growth conditions

The bacterial strains used in this study are described in Table 2. *Escherichia coli* strains were grown at 37°C on LB Lennox (LBL) agar or in LBL broth (Difco). *B. mallei* strains were grown at 37°C in LBL broth or on LBL agar supplemented with 4% glycerol. When appropriate, antibiotics were used at the following concentrations: 10 μg ml^−1^ gentamicin or 15 μg ml^−1^ polymyxin B for *E. coli* and 5 μg ml^−1^ gentamicin for *B. mallei*. Bacterial stocks were maintained at −80°C as 20% glycerol suspensions. All studies utilizing viable *B. mallei* were conducted under biosafety level three containment conditions.

**Table 2:** Bacterial strains, plasmids and primers used in this study.

Strain, plasmid or primer	Description	Source or reference
Strain		
*E. coli*		
TOP10	High efficiency transformation: Gm^s^	Invitrogen
S17-1	Mobilizing strain; transfer genes of RP4 integrated in chromosome: Gm^s^, Pm^s^	Simon *et al.* (1983)
*B. mallei*		
ATCC 23344	Type strain; isolated in 1944 from a human case of glanders: Gm^s^, Pm^r^	Yabuuchi *et al.* (1992)
GM3773	ATCC 23344::pGMwcbB: Gm^r^, Pm^r^	This study
Plasmids		
pGSV3	Mobilizable suicide vector: Gm^r^	DeShazer *et al.* (2001)
pGMwcbB	pGSV3 containing a 547 bp PCR fragment internal to *wcbB*: Gm^r^	This study
Primers		
wcbB-iF	5′-CCCAACGGTCTCAAATTCGATATTCAGGCTAGCCGCGC-3′	This study
wcbB-iR	5′-CCCAACGGTCTCGCTAGTGACGTTCTCGCATTCCCAGCCTCG-3′	This study
wcbB-up	5′-ACGAAGTCCGCCGTGTTTCGATCAGC-3′	This study
MB1ori-P1	5′-GAAGATCCTTTGATCTTTTCTACGG-3′	This study

### Recombinant DNA techniques

DNA manipulations were performed using standard methods. Polymerase chain reaction (PCR) and restriction enzyme digested products were purified using a QIAquick Gel Extraction Kit (Qiagen). The plasmids described in Table 2 were purified using a QIAprep Spin Miniprep Kit (Qiagen). Chromosomal DNA was isolated using a Wizard™ Genomic DNA Isolation Kit (Promega). Chemically competent *E. coli* were transformed using standard methods.

### Mutant construction

The *B. mallei* GM3773 CPS mutant was constructed essentially as previously described (DeShazer *et al*., 2001; Burtnick *et al*., 2002). Briefly, a 547 bp fragment internal to the *wcbB* allele was PCR amplified from purified *B. mallei* ATCC 23344 chromosomal DNA using the wcbB-iF and wcbB-iR primer pair (Table 2); the BsaI/EcoRI and BsaI/NheI restriction sites in the linker regions are underlined. The amplified product was then cloned into pGSV3. The resulting suicide plasmid, pGMwcbB, was mobilized into ATCC 23344 via conjugative mating using *E. coli* S17-1. Site specific recombination of the plasmid into the ATCC 23344 chromosome was verified via PCR using the wcbB-up and MB1ori-P1 primer pair (Table 2).

### Lipopolysaccharide purification and lipid a isolation

LBL broth inoculated with *B. mallei* ATCC 23344 or GM3773 was incubated overnight at 37°C with vigorous shaking. Cell pellets were subsequently obtained by centrifugation and extracted using a modified hot aqueous-phenol procedure (Perry *et al*., 1995). Following extraction, the resulting phenol and aqueous phases were combined and dialysed in distilled water to remove the phenol. The dialysates were then clarified by centrifugation and concentrated by lyophilization. The crude preparations were solubilized (20 mg ml^−1^) in RD buffer [10 mM Tris-HCl (pH 7.5), 1 mM MgCl_2_, 1 mM CaCl_2_, 50 μg ml^−1^ RNase A and 50 μg ml^−1^ DNase I] and incubated for 3 h with shaking at 37°C. Proteinase K was then added to a final concentration of 50 μg ml^−1^ and the digests were incubated for an additional 3 h at 60°C. The enzymatic digests were clarified by centrifugation and the supernatants filter sterilized. LPS was then isolated from the supernatants as precipitated gels following three rounds of ultracentrifugation at 100 000 *g* and 4°C. After the final spin, the gel-like pellets were resuspended in pyrogen-free water and lyophilized. To remove contaminating phospholipids, the lyophilized LPS samples were repeatedly extracted with 90% EtOH. Lipid A was isolated from the purified LPS samples via mild acid hydrolysis. Briefly, LPS was hydrolysed for 4 h at 95°C in 0.1 M NaOAc (pH 4.5). The lipid A fraction was then removed by extraction with chloroform:methanol (2:1) and dried under a gentle stream of air and lyophilized.

### SDS-PAGE, immunoblotting and ELISA analyses

Purified samples were solubilized in 1× SDS-PAGE sample buffer and heated to 100°C for 5 min prior to electrophoresis on 12% Express Gels (ISC BioExpress). The LPS was visualized by silver staining as previously described (Fomsgaard *et al*., 1990). Immunoblot analysis was performed as previously described (Burtnick *et al*., 2002). ELISA were performed essentially as previously described (DeShazer *et al*., 1998). The primary antibodies used in this study were the O-antigen specific 3D11 monoclonal antibody (mAb; Research Diagnostics) and the CPS specific MCA147 mAb (Reckseidler-Zenteno *et al*., 2005). The secondary antibodies used were anti-mouse IgG or IgM horse radish peroxidase conjugates (Sigma).

### Glycosyl composition analysis

Per-*O*-trimethylsilyl (TMS) derivatives of monosaccharide methyl glycosides produced from the acidic methanolysis of the lipid A samples were analysed by gas chromatography/mass spectrometry (GC/MS). The methyl glycosides were prepared by heating the dried lipid A samples in acidified methanol (1 M HCl) at 80°C for 18–22 h. Following this, the samples were re-*N-*acetylated with pyridine and acetic anhydride in order to facilitate the detection of amino sugars. The samples were then per-*O*-trimethylsilylated via treatment with Tri-Sil (Pierce) at 80°C for 30 min. GC/MS analysis of the TMS methyl glycosides was performed on a HP 5890 GC interfaced with a 5970 MSD using a All Tech EC-1 fused silica capillary column (30 m × 0.25 mm ID).

### Fatty acid analysis

Methyl ester derivatives of the fatty acids generated by acidic methanolysis of the lipid A samples, were dried, resuspended in hexane and analysed by GC/MS to determine total fatty acid content. In order to detect *O*-linked fatty acids, the lipid A samples were suspended in alkaline methanol (0.25 M NaOMe), incubated overnight at 37°C and then neutralized with an equal volume of 0.25 M HCl. The fatty acid methyl esters were then extracted with 0.15 volumes of chloroform and analysed by GC/MS after a partial drying under a gentle stream of dry air. In addition to using the All Tech EC-1 fused silica capillary column, GC was also performed using a Supelco SP 2380 capillary column equipped with a FID detector and a bimodal temperature gradient starting at 130°C and increasing at 4°C per minute to 190°C followed by a 1 min hold at 190°C and a 17°C per minute gradient to 260°C and 3 min hold.

### Mass spectrometry

Matrix-assisted laser desorption/ionization time-of-flight mass spectrometry (MALDI-TOF-MS) was performed in the negative-ion mode on a Voyager DE-STR mass spectrometer (Applied Biosystems). Lipid A samples in chloroform:methanol (2:1) were mixed on target with 2,5-dihydroxybenzoic acid (500 mM in anhydrous methanol) via the dried droplet method. The following parameters were used: N2 laser, 337 nm, accelerating voltage 20 kV with delayed extraction and mass range of 500–5000. MALDI-TOF spectrometry was also performed on an Applied Biosystems 4700 Proteomics Analyzer in the positive ion reflector mode.

### Cell culture

Human embryonic kidney 293 (HEK 293) cells transfected with human MD2-CD14 (HEK 293-hMD2-CD14; Invivogen), human TLR2/CD14 (HEK 293-hTLR2/CD14; Invivogen) or human TLR4/MD2-CD14 (HEK 293-hTLR4/MD2-CD14; Invivogen) were maintained at 37°C with 5% CO_2_ in Dulbecco's modified Eagle's medium (Invitrogen) supplemented with 10% heat-inactivated (HI) fetal bovine serum (FBS), 10 μg ml^−1^ Blasticidin S and 50 μg ml^−1^ Hygromycin B (Invivogen). Prior to stimulation with LPS, the HEK 293 cell lines were transferred into 24-well tissue culture plates at 5 × 10^5^ cells well^−1^. U-937 and THP-1 human monocytes (ATCC) were maintained at 37°C with 5% CO_2_ in RPMI 1640 medium (Invitrogen) supplemented with 10% HI FBS and 100 μg ml^−1^ Normocin™ (Invivogen). To differentiate the monocytes into macrophage-like cells, the U-937 and THP-1 cells were transferred into 24-well tissue culture plates at 1 × 10^5^ cells well^−1^ and incubated at 37°C with 5% CO_2_ for 36 h in the presence of 50 ng ml^−1^ phorbol myristate acetate. Prior to stimulation with LPS, the differentiated cells were washed with Hanks balanced salt solution (Invitrogen).

### Human monocyte-derived macrophage and dendritic cells

Human peripheral blood mononuclear cells were isolated from buffy coats by centrifugation through a Ficoll-Paque Plus density gradient (Amersham Pharmacia Biotech). Cells were enriched for monocytes (CD14+ cells) using a RossetteSep Monocyte Enrichment Kit (Stem Cell Technologies). Macrophages (MDM) were obtained by resuspending the monocytes (1 × 10^6^ cells ml^−1^) in MΦ medium (RPMI 1640, 10% HI FBS, 50 ng ml^−1^ recombinant human M-CSF; Peprotech) and then culturing them at 37°C with 5% CO_2_ for 7 days with the addition of fresh cytokine on day three. On day seven, the adherent cells were washed once with phosphate-buffered saline (PBS) and then incubated on ice for 20 min in PBS. The cells were then harvested by gentle scraping and centrifugation (500 *g*), resuspended in MΦ medium and transferred into 24-well tissue culture plates at 1 × 10^5^ cells well^−1^ prior to LPS stimulation. Monocyte-derived dendritic cells (MDDC) were isolated and the CD14 blocking studies were performed as previously described (Shannon *et al*., 2005).

### Lipopolysaccharide stimulation assays and cytokine quantification

Prior to use, the *E. coli* O55:B5 (Sigma) and *B. mallei* GM3773 LPS samples were repeatedly extracted with 90% EtOH to remove any contaminating phospholipids (Zughaier *et al*., 2005). LPS stocks were prepared in pyrogen free water (Cambrex) and were quantified on a w/v basis. HEK 293 cell lines were stimulated for 6 h with varying concentrations (0.1–100 ng ml^−1^) of GM3773 or O55:B5 LPS following which the culture supernatants were harvested and assayed for IL-8 production using Human IL-8 Chemiluminescent ELISA Kits (Endogen). THP-1, U-937, MDM and MDDC cells were stimulated for 24 h with 10 ng ml^−1^ of GM3773 or O55:B5 LPS following which the culture supernatants were harvested and assayed for TNF-α, IL-6, RANTES and IP-10 production using human SearchLight Custom Multiplex Arrays (Pierce).
